# β-galactosidase GALA from *Bacillus circulans* with high transgalactosylation activity

**DOI:** 10.1080/21655979.2021.1988370

**Published:** 2021-11-01

**Authors:** Yaru Yan, Weishi Guan, Xiaoyi Li, Kaier Gao, Xinxin Xu, Bo Liu, Wei Zhang, Yuhong Zhang

**Affiliations:** a Biotechnology Research Institute, Chinese Academy of Agricultural Sciences, Beijing, China; b College of Letters and Science, University of California, Santa Barbara, Santa Barbara, California, USA

**Keywords:** Galacto-oligosaccharides, β-Galactosidase, transgalactosylation, *Bacillus circulans*, *Pichia pastoris*, response surface methodology

## Abstract

β-galactosidase catalyzes lactose hydrolysis and transfers reactions to produce prebiotics such as galacto-oligosaccharides (GOS) with potential applications in the food industry and pharmaceuticals. However, there is still a need for improved transgalactosylation activity of β-galactosidases and reaction conditions of GOS production in order to maximize GOS output and reduce production costs. In this study, a β-galactosidase gene, *galA*, from *Bacillus circulans* was expressed in *Pichia pastoris*, which not only hydrolyzed lactose but also had strong transgalactosylation activity to produce GOS. Response surface methodology was adopted to investigate the effects of temperature, enzyme concentration, pH, initial lactose concentration, and reaction time on the production of GOS and optimize the reaction conditions for GOS. The optimal pH for the enzyme was 6.0 and remained stable under neutral and basic conditions. Meanwhile, GALA showed most activity at 50°C and retained considerable activity at a lower temperature 30–40°C, indicating this enzyme could work under mild conditions. The enzyme concentration and temperature were found to be the critical parameters affecting the transgalactosylation activity. Response surface methodology showed that the optimal enzyme concentration, initial lactose concentration, temperature, pH, and reaction time were 3.03 U/mL, 500 g/L, 30°C, 5.08, and 4 h, respectively. Under such conditions, the maximum yield of GOS was 252.8 g/L, accounting for approximately 50.56% of the total sugar. This yield can be considered relatively high compared to those obtained from other sources of β-galactosidases, implying a great potential for GALA in the industrial production and application of GOS.

## 1. Introduction

β-galactosidase (EC 3.2.1.23), also known as lactase, catalyzes two different types of reactions, namely hydrolysis and transgalactosylation [1]. It hydrolyzes lactose into glucose and galactose, which has been broadly exploited in the food industries, with alleviating lactose maldigestion and enhancing sweetness, flavor, and solubility of lactose [2]. On the other hand, transgalactosylation reaction is commonly applied to produce lactose-based prebiotics such as galacto-oligosaccharides (GOS) which have beneficial effects on human health [3]. GOS is a low-caloric oligosaccharide composed of 2 ~ 10 galactose units with one terminal glucose residue. When using lactose as the substrate, β-galactosidase cuts the β-1,4 glycosidic bond and connects the free galactosyl group with other glycosidic receptors by glycosidic bond such as β-1,3, β-1,4, or β-1,6 [4], and ultimately results in the formation of GOS. Although GOS cannot be absorbed by the body, they can modulate colonic flora by stimulating beneficial bacteria such as *Bifidobacterium* and *Lactobacillus*, and inhibiting less desirable bacteria, providing favorable benefits on immunological stimulation, mineral absorption, and the prevention of colon cancer [5,6]. Therefore, GOS has bright prospects in the field of functional foods and pharmaceuticals.

There are various GOS production methods, including extraction from natural raw materials, chemical synthesis, acid hydrolysis, direct fermentation synthesis, and enzymatic synthesis [1,50]. As an environment-friendly biocatalyst, β-galactosidase has been widely studied for its recognized advantages. The ratio between lactose hydrolysis and GOS synthesis crucially depends on the concentration of lactose, temperature of the reactor, and intrinsic properties of enzymes De [7, 8]. Therefore, it is of importance to optimize the conditions for GOS synthesis and to discover β-galactosidases with high transgalactosylation capacity.

β-galactosidases are widely distributed in microorganisms, plants, and animal tissues [9]. Among them, β-galactosidase from microbial sources has the greatest potential for industrial applications mainly due to their easy handling, greater catalytic activity, and high production yield [10]. And so far, most β-galactosidases of commercial interest have been isolated from *Kluyveromyces lactis, Kluyveromyces fragilis, Candida kefyr, Thermotoga maritima, Sulfolobus solfataricus, Aspergillus niger, Aspergillus oryzae,* and *Bacillus circulans* [11,12]. In particular, the *Bacillus circulans* β-galactosidase synthesizes GOS with a major presence of β (1→4) bonds [13,14] and gives rise to a notable GOS yield (approximately 49% w/v, starting with 400 g/L lactose) [15,16].

Many studies have been conducted in pursuit of a higher production of GOS. When the amino acids of the thermostable recombinant β-galactosidase from *Halothermothrix orenii* were altered from Phe417 to Tyr417 and from Tyr296 to Phe296, the GOS yield from lactose increased by approximately 46% and 33.6%, respectively, where the reactions were performed at 70°C with an initial lactose concentration of 300 g/L in sodium phosphate buffer (pH 6.0) [17,51] performed multiple sequence alignment to identify the potential mutation sites associated with increased GOS formation. They found that Phe426, Phe401, and Phe441 were conserved in β-glycosidase of *Pyrococcus furiosus, Thermus thermophilus*, and *Sulfolobus solfataricus*, respectively, and therefore mutant F441Y was constructed to investigate GOS production. Under the optimal conditions, yields of GOS for wild-type enzyme and mutant F441Y could reach 50.9% and 61.7%, respectively. Additionally, β-galactosidase was immobilized on chitosan-coated magnetic nanoparticles to produce GOS from lactulose. The stability of β-galactosidase was significantly improved, and the maximum yield of GOS was 17% at the initial lactulose concentration of 2.34 M for 36 h of reaction [18]. Nonetheless, it is still imperative to find new β-galactosidases with higher transgalactosylation activity and to optimize reaction conditions of GOS production in order to maximize GOS output and reduce production costs.

We hypothesized that β-galactosidase expressed in *P. pastoris* would have higher transgalactosylation activity and GOS yields. Therefore, the aim of this study was to characterize the β-galactosidase from *B. circulans* which featured quite high transgalactosylation properties and to optimize the main reaction parameters (pH, temperature, time, initial lactose concentration, and the amount of β-galactosidase) of transgalactosylation activity to produce more GOS.

## 2. Materials and methods

### 2.1. Strains, plasmids, and medium

*Escherichia coli* (*E. coli*) Trans10 (TransGen, Beijing, China) and *P. pastoris* GS115 (Invitrogen, Carlsbad, CA, USA) were used as the gene cloning and expression hosts, respectively. The plasmid pUC57-*galAm* harboring the gene of β-galactosidase from *B. circulans* and His-tag was optimized and synthesized by the GenScript Corporation (Nanjing, China). The GenBank accession number of the β-galactosidase gene is MN443117. The *P. pastoris-E. coli* shuttle expression vector pPICZαA (Invitrogen, Carlsbad, CA, USA) was used for gene cloning and expression in those two hosts. All medium, including Luria-Bertani (LB) medium, yeast peptone dextrose (YPD) medium, buffered glycerol-complex (BMGY) medium, buffered methanol-complex (BMMY) medium, and minimal methanol (MM) medium, were prepared according to the instructions in the Pichia expression kit (Invitrogen, Carlsbad, CA, USA).

### 2.2 Chemicals

5-Bromo-4-chloro-3-indolyl-β-D-galactopyranoside (X-gal), trichloroacetic acid (TCA), D-β-galactose, D-glucose, O-nitrophenyl-β-D-galactopyranoside (*o*NPG), Zeocin, and bovine serum albumin (BSA) were purchased from Sigma Aldrich (St. Louis, USA). Amino acid-free yeast nitrogen source (YNB) was derived from Difco (Detroit, MI). Enzymes and protein ladders were purchased from Thermo Scientific (Rockford, IL, USA).

### 2.3 *Construction of recombinant plasmid pPICZαA-*galAm

The strains with plasmids pUC57-*galAm* and pPICZαA were cultivated at 37°C overnight in LB medium containing 50 mg/L ampicillin and 25 mg/L zeocin, respectively. The plasmids were extracted using the DNA Extraction Kit (TIANGEN Cor., Beijing, China) according to the manufacturer’s guide and then treated with endonucleases FastDigest *Eco*RI and *Not*I at 37°C for 3 h. The digested products were purified using the DNA Gel Extraction Kit (TIANGEN Cor., Beijing, China). The resultant two fragments were ligated in the presence of T4 DNA ligase at 25°C for 1 h, and the recombinant plasmids were transformed into *E. coli* Trans10 for gene cloning. The strain with expression plasmid pPICZαA-*galAm* was cultivated at 37°C and the plasmid was extracted. Finally, the plasmids were linearized with *Sac*I and transformed into *P. pastoris* GS115 competent cells by electroporation.

### 2.4 Positive transformant screening

Transformants were cultivated on YPD plates containing 300 mg/L zeocin at 28°C for 48 h, and then individual positive transformants were placed in 48-well microtiter plates containing 500 µL of BMGY medium, and then grown at 28°C for 48 h at 200 rpm in a shaker incubator. BMGY medium was discarded by centrifugation and the transformants were further cultivated in BMMY at 28°C for 72 h where 1% methanol was added every 24 h. The enzyme solutions were harvested by centrifugation, and the enzyme activity was measured. A transformant with the highest enzyme activity was finally selected for the target strain.

### 2.5 Protein expression, purification, and analysis

The target strain was cultured in 2 ml of YPD containing 300 mg/L of zeocin for 48 h and then inoculated and induced in 50 ml of BMGY at 28°C for 48 h in shaking flasks. The cells were harvested by centrifugation (5000 rpm for 5 min) and then grown in 25 ml of BMMY with methanol induction (1.0% v/v) at 28°C for 72 h. The supernatant was collected by centrifugation (5000 rpm for 5 min) and concentrated using a 10 kDa molecular weight cutoff membrane, and dialyzed in 50 mL of buffer A (20 mM phosphate–citrate buffer, pH 6.0). The retentate was applied on a Histrap HP column (1 mL) that was pre-equilibrated with buffer on an automated fast protein liquid chromatography (FPLC) system (Äkta Purifier, GE Healthcare, USA). The recombinant β-galactosidase was eluted using a linear gradient of imidazole from 0 to 0.5 M. The fractions showing β-galactosidase were further purified via molecular sieve chromatography (GE Healthcare) with buffer A as the mobile phase. Protein concentration was measured with the BCA Protein Assay Kit (CWBIO, Beijing, China). Sodium dodecyl sulfate-polyacrylamide gel electrophoresis (SDS-PAGE) and Native-PAGE analysis were performed using a 12% running gel. Protein bands were stained using either Coomassie Blue or X-gal as a substrate for active staining. In order to obtain more protein, the high-cell-density fermentation was performed according to the Pichia fermentation guidelines (Invitrogen, Carlsbad, CA, USA) in a 2 L bioreactor, followed by protein purification using the same method as described above.

### 2.6 Enzyme activity assay

β-galactosidase activity was determined using the *o*NPG method [19]. The reaction was initiated by adding 200 μL of dissolved enzyme solution to 800 μL of 0.25% *o*NPG in buffer A, which was incubated at 45°C for 15 min. The reaction was stopped by the incorporation of 1 mL of 10% TCA. Then, 2 mL of 10% Na_2_CO_3_ was added to stabilize the color of the reaction. The released o-nitrophenol was determined by measuring the absorbance at 420 nm using a Benchmark Plus Microplate Spectrophotometer (Bio-Rad, California, USA). One unit (U) of enzyme was defined as the amount of the enzyme that liberates 1 μmol of o-nitrophenol from *o*NPG per minute under the assay conditions.

### 2.7 Effects of temperature and pH on enzyme activity

The optimum temperature of the β-galactosidase was determined at temperatures ranging from 20°C to 65°C for 0.25% *o*NPG in buffer A. The enzyme activity at optimum temperature was defined as 100%. For thermal stability measurements, each enzyme dissolved in buffer A at a final concentration of 0.3 mg/mL was pre-incubated for 1 h at temperatures ranging from 20°C to 65°C, and the remaining activity was assayed using *o*NPG as a substrate, as described above. The ideal pH for the β-galactosidase was measured using dissolved *o*NPG as substrates in 50 mM phosphate–citrate buffer at pH values ranging from 4.5 to 9.5 at 45°C. For pH stability measurements, the enzyme was incubated at 30°C in 50 mM phosphate–citrate buffer at pH 4.5–9.5 for 120 min in the absence of the substrate at the final concentration of 0.3 mg/mL. The remaining activity was then assayed using dissolved *o*NPG in buffer A as a substrate by the method described above. The enzyme activity at optimum pH was defined as 100%.

### 2.8 Transgalactosylation reaction analysis

Transgalactosylation efficiency of the β-galactosidase was analyzed by high performance liquid chromatography (HPLC) [20,21]. Briefly, all reactions were performed at 30–50°C in conical flasks on an orbital shaker set at 200 rpm. The reaction mixture was composed of 440 μL of 200–500 g/L lactose in 0.1 M phosphate–citrate buffer (pH 5–7) and 60 μL of 3–12 U/mL enzyme (measured in the presence of *o*NPG). Samples were taken at different times and then heated at 100°C for 10 min to stop the reaction by the inactivation of the enzyme. In order to collect the supernatant from the reaction mixture, samples were centrifuged at 10,000 rpm for 10 min and filtered through 0.2 μm syringe filters. Seven hundred μL of the reaction mixture diluted with deionized water was analyzed by HPLC. Control samples without enzymes were prepared simultaneously by exposure to the same temperature treatment; no galacto-oligosaccharide reaction products were detected in these control samples. All experiments were carried out in duplicates and the average concentration values for the products obtained are presented in the figures; all standard deviations were less than 5%. The amounts of extra lactose and reaction products galactose and glucose were determined by HPLC with a 6.5 × 300 mm Sugar-Pak I column (Waters) and 50 mg/ml CaNa_2_-EDTA buffer as the mobile phase (Choi, et al. 2020). The concentration of GOS was calculated from the concentrations of lactose, galactose, and glucose using the equation:

GOS = Ci-Cf-galactose-glucose

The lactose conversion and GOS yield were calculated using the following equations.

Lactose conversion = (Ci-Cf)/Ci×100

GOS yield = Cp/Ci×100

where Ci and Cf are the initial and final concentrations of lactose, respectively, and Cp is the concentration of GOS [1].

In addition, thin-layer chromatography (TLC) was used to determine the composition of GOS mixture, following previously described methods [22,23]. The analysis of GOS and other products was accomplished on a precoated 10 × 10 cm high-performance lamina chromatography (HPTLC) silica gel 60 plate (Merck KGaA, Germany). 5 µL of 5% lactose (m/v), 5% galactose (m/v), 5% glucose (m/v), 20% GOS sample, and 20% commercial GOS product (m/v) were pipetted on the plate. The linear ascending development of the plate was accomplished in a pre-saturated glass chamber (21× 9× 21 cm) with solvent consisting of n-butyl alcohol:isopropanol: water (3:12:4, v/v/v) for 1 h at room temperature. The plate was air-dried at room temperature for 10 min. The spots were visualized by fully submerging the plate into sulfuric acid ethanol (30%, v/v) for 10 s. The plate was dried in an oven set at 100°C for 10 min.

### 2.9 Optimization of the synthesis of high GOS

Rational optimization of the synthesis of GOS catalyzed by the β-galactosidase was performed by response surface methodology (RSM) [24,25,26,52]. Firstly, a factorial experimental design was carried out to assess the simultaneous and combined effect of the main reaction parameters affecting GOS production using Design Expert version 8 software (Stat-Ease Inc., Minneapolis, MN, USA) [27]. Such parameters include temperature, enzyme concentration, pH, initial lactose concentration and reaction time [28]. A 5^3^ factorial experimental design (five factors at three levels) was carried out using a Plackett–Burman design (PBD), to determine the significant influencing factor and the optimum reaction conditions for GOS production. The central point was repeated three times to determine the variability of the results and to assess the experimental error. The selected response for the analysis was GOS yield. The selection of the levels for each factor was based on either experimental results or previously reported data.

## 3. Result and discussion

The aim of this study was to find higher transgalactosylation activity β-galactosidases and increase GOS yields by optimizing reaction conditions. The β-galactosidase GALA from *B. circulans* was expressed in *P. pastoris* and purified for further characterization. The optimal pH and temperature of GALA were examined based on *o*NPG converting activity. Moreover, the response surface methodology was used to optimize the main parameters including pH, temperature, time, enzyme concentration, and initial lactose concentration of transgalactosylation reaction to produce more GOS.

### 3.1 Protein expression and purification of GALA

Ten transformants were chosen to be induced with 1% methanol and the supernatants were used in the measurement of enzyme activity. Clone #2 demonstrated the highest activity and was chosen for further analyses (Figure S1).

**Figure 1.**
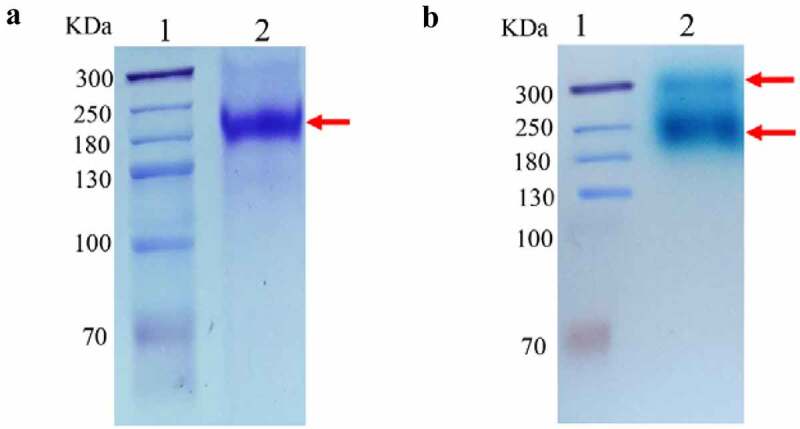
Protein analysis of GalA: (a) Sodium dodecyl sulfate-polyacrylamide gel electrophoresis(SDS-PAGE) of purified β-galactosidase. Lane 1, molecular weight marker; lane 2, purified enzyme sample treated in SDS buffer at 100°C for 10 min, with Coomassie brilliant blue R250 staining. (b) Native polyacrylamide gel electrophoresis(Native-PAGE) of the culture supernatant from *Pichia pastoris*. Lane 1, molecular weight marker; lane 2, crude enzyme sample, with X-gal staining

The target strain Clone #2 was cultured and induced in shaking flasks, and the hydrolysis activity of the supernatant measured with *o*NPG was 0.5 U/mL, which was increased to 1.4 U/mL following the high-cell-density fermentation. The obtained GALA protein was predicted to encode 1703 residues with a molecular weight of 190 kDa (analyzed by Vector NTI 11.5.1). As shown in Figure 1a, SDS-PAGE analysis of the purified GALA showed that the band size was consistent with the theoretical molecular weight of 190 kDa. Active staining on Native-PAGE with X-gal yielded two bands showed β-galactosidase activity (Figure 1b), indicating that glycosylation occurred during post-translational modification in *P. pastoris*.

*P. pastoris*, an established protein expression host, is mainly applied for the production of biopharmaceuticals and industrial enzymes. This methylotrophic yeast is a distinguished production system for its growth to very high cell densities, the available strong and tightly regulated promoters, and the options to produce gram amounts of recombinant protein per liter of culture both intracellularly and in secretory fashion [29]. In this study, a β-glucosidase from *B. circulans* was expressed in *P. pastoris*. However, the expression level of the enzyme was still not ideal, and strategies are needed to further improve the expression. In addition to the yeast expression system, the recombinant β-galactosidase gene has been successfully expressed in *Escherichia coli, Lactobacillus plantarum, Lactococcus lactis,* and *Saccharomyces cerevisia*e [23]. However, the recombinant expression of β-galactosidases in *E. coli, Lactobacillus,* and *Lactococcus* are usually cytoplasmic, making the purification tedious and costly [12]. Besides heterogeneous expression, many of β-galactosidases were produced by native fermentation such as *Alicyclobacillus vulcanalis* [30], *Kluyveromyces lactis* De [31] and *Aspergillus terreus* [2]. However, some typical problems exist during their production, e.g., the fermentation may contain other endogenous proteins leading to complex downstream processing and high economic costs.

### 3.2 Effect of temperature and pH on enzymatic hydrolysis activity and stability

**Figure 2.**
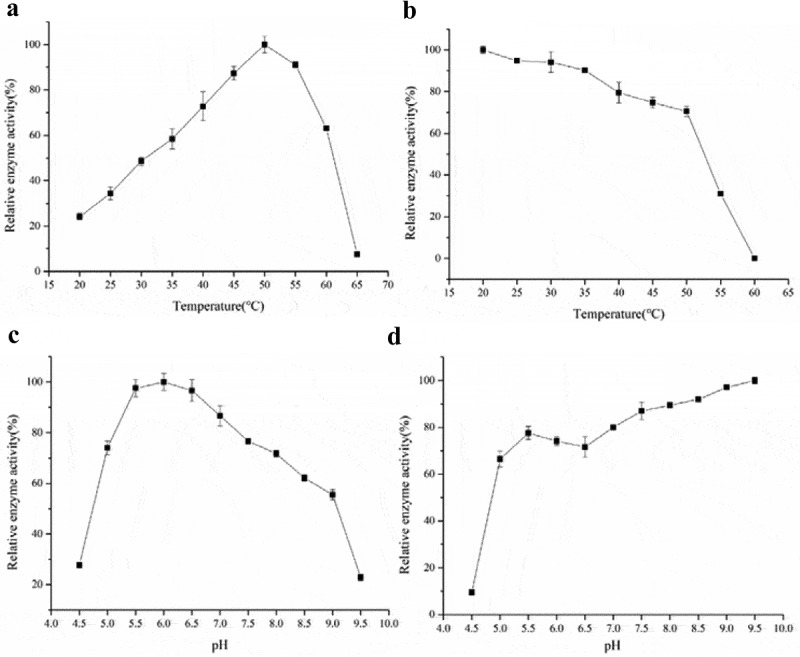
The effect of temperature and pH on purified β-galactosidase. (a) The optimal temperature for the purified β-galactosidase. The enzyme activity assay was carried out at different temperatures ranging from 20 to 65°C in 5-unit increments. (b) The heat tolerance of the purified β-galactosidase. The enzyme activity assay was carried out at temperatures ranging from 20 to 60°C. (c) The optimal pH for the purified β-galactosidase. (d) The pH tolerance of the purified β-galactosidase. The enzyme activity assay was carried out in buffers with different pH values ranging from 4.5 to 9.5 in 0.5-unit increments. The experiments were carried out three times using ONPG as the substrate, and the data are presented as Mean ± SD

Results showed that the optimal temperature of the recombinant β-galactosidase was 50°C (Figure 2a). However, the enzyme still retained considerable activity at lower temperatures 20–30°C, suggesting that the enzyme could work under milder conditions, which can reduce energy consumption and environmental pollution. Figure 2b shows that the recombinant β-galactosidase was quite thermally stable. The residual hydrolysis activity was more than 80% at 20°C–40°C, and about 50% after incubation at 55°C for 60 min.

As shown in Figure 2c, the recombinant enzyme demonstrated significant *o*NPG hydrolysis in the pH range of 5.5–6.5 (optimal at 6.0), but the enzymatic activity decreased dramatically at pH less than 5.0 and higher than 9.0, which aligns well with the natural pH of milk. The recombinant enzyme worked predominantly in basic conditions, as reflected by the results that the residual hydrolysis activity was found to be more than 80% in alkaline region (pH 8.0–9.5) (Figure 2d). These results were similar to those reported in the literature for other β-galactosidases, in response to temperature and pH (Table 1). For example, the recombinant β-galactosidase from the Antarctic bacterium *Alteromonas* sp. ANT48 cloned and expressed in *E. coli* showed most activity at 50°C and retained more than 80% of its initial activity below 40°C [23,24–26]. A cold active β-galactosidase from *Planococcus* sp-L4 was optimized for expression in *P. pastoris* [9]. The recombinant enzyme displayed significant activity at pH 6.5 but the activity decreased dramatically at pH below 5.5 and above 8. Moreover, the β-galactosidase from *B. subtilis* was expressed in *E. coli*, and displayed an optimum activity at pH 6.5 and 40°C [32,33]. The optimum temperature was 10°C lower than in the present study, which may be caused by the lack of post-translational modifications in the *E. coli* expression system.

**Table 1.** Literature review on GOS production from lactose by microbial β-galactosidases

Enzyme source	Expression system	Temp. (°C)	pH	Lactose (g/L)	GOS (g/L)	Y_GOS_ (%)	Reference
*Bacillus circulans*	*Pichia pastoris*	30	5.1	500	252.8	50.56	The current study
*Streptococcus thermophilus*	*Lactobacillus plantarum*	50	6.5	205	102.5	50	[5]
*Bifidobacterium*	*Escherichia coli*	45	8.5	360	91.1	25.3	[44]
*Aspergillus oryzae*	*Aspergillus oryzae*	40	4.5	400	107	26.8	[53]
*Kluyveromyces lactis*	*Kluyveromyces lactis*	40	7	400	177	44	[16]
*Lactococcus lactis*	*Lactococcus lactis*	50	7	500	175	35	[54]
*Bacillus circulans*	*Escherichia coli*	40	6	400	253.6	63.4	[55]
*Alteromonas sp. ANT48*	*Escherichia coli*	40	7			30.9	[23]
*Kluyveromyces lactis*	*Kluyveromyces lactis*	40	8		140.1	35	[56]
*Halothermothrix orenii*	*Escherichia coli*	70	6	300	118	39.3	[17]
*Halothermothrix orenii*	*Escherichia coli*	70	7	400	185	46.25	[17]
*Bifidobacterium longum EK3*	*Escherichia coli*	50	6.5	200	66.8	33.4	[57]
*Lichtheimia ramosa*	*Lichtheimia ramosa*	50	6	150	16.5	0.11	[58]
*Rhizomucor pusillus*	*Rhizomucor pusillus*	50	6	150	25.5	0.17	[58]
*Lactobacillus sakei*	*Escherichia coli*	37	6.5	215	88	41	[45]
*Lactobacillus plantarum*	*Lactobacillus plantarum*	37	6.5	205	84	41	[46]
*Lactobacillus delbrueckii subsp*	*Lactobacillus plantarum*	30	6.5	205	101	49.5	[47]
*Lactobacillus pentosus*	*Lactobacillus pentosu*	30	6.5	208	65	31	[48]
*Bacillus circulans*	*Bacillus circulans*	40	6	525	204	39	[40]
*Sporobolomyces singularis*	-	55	5	600	242	40	[46]
*Streptococcus thermophilus*	*Lactobacillus plantarum*	50	6.5	205	103	50	[5]
*L.delbrueckii subsp*	*Lactobacillus plantarum*	50	6	205	103	50	[49]

### 3.3 Enzymatic synthesis of galacto-oligosaccharides

**Figure 3.**
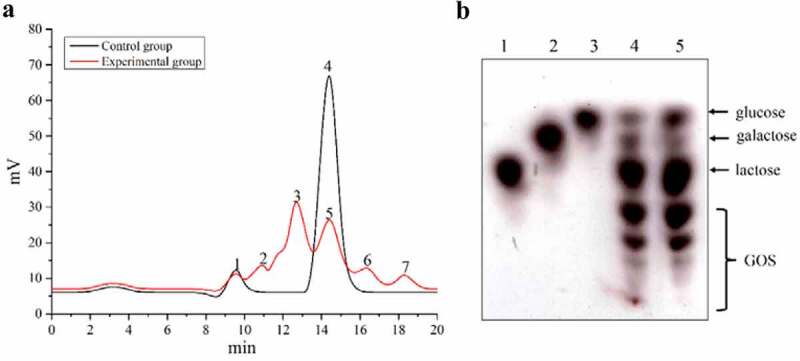
Analysis of GOS production achieved by enzymatic transgalactosylation activity of the β-galactosidase, using lactose as the substrate. (a) The presence of the different sugars in the reaction mixture was analyzed by HPLC: (1) solvent; (2, 3) GOS product; (4, 5) lactose; (6) glucose; (7) galactose. (b) High performance thin layer chromatogram of 20% galacto-oligosaccharides sample on precoated silica gel. 5 µL of each sample was spotted on the plate using micropipettes: (1) 5% lactose; (2) 5% galactose; (3) 5% glucose; (4) GOS product obtained in this study, (5) commercial GOS product

The reaction mixture was analyzed using HPLC and TLC to detect the presence of different sugars. As shown in Figure 3a, the formation of GOS could be significantly seen under standard conditions. During hydrolysis, aside from lactose (peaks 4 and 5), galactose (peak 7), glucose (peak 6), and GOS (peaks 2 and 3) were also formed as a result of transgalactosylation catalyzed by the enzyme, compared with control group without the enzyme, which was consistent with TLC result. In addition, the relative proportions of all kinds of oligosaccharides in the GOS generated in this study were close to commercial GOS products (Figure 3b), which further demonstrated that GALA is a potential candidate for catalyzing the production of GOS. The results in Figure 3 clearly indicate that the GOS produced by β-galactosidase was a mixture of transgalactosylated oligosaccharides, unreacted lactose, glucose, and galactose. Extensive research has been carried out on the components of GOS products despite inconsistent results. Simultaneous synthesis and purification of GOS from lactose was conducted using a combi-catalyst, which was composed by β-galactosidase and *S. cerevisiae* cell aggregates to improve the purity of GOS [13] [34], showed that GOS mixture contained distinct oligosaccharides varying in glycosidic linkages with a polymerization degree of 2–8 [34]. The glycosidic bond structures of GOS synthesized by β-galactosidase from *L. plantarum* were β-D-Galp-(1→6)-D-Lac, β-D-Galp-(1→3)-D-Lac, and β-D-Galp-(1→6)-DGlc [35]. The majority of the produced GOS by a novel thermophile β-galactosidase was trisaccharides, while low concentrations of disaccharides and tetrasaccharides were also detected [36]. Taken together with these studies, more work is still needed to identify and purify GOS in the future.

### 3.4 Optimization of the synthesis of GOS by response surface methodology

Five parameters were chosen to optimize the synthesis of GOS using a response surface methodology designed by Design Expert software (version 8). The three selected levels for each factor in the design were as follows: pH 5, pH 6, and pH 7, 300, 400, and 500 g/L for initial lactose concentration, 4, 10, and 20 h for reaction time, 30, 40, and 50°C for temperature, and 3, 6, and 12 U/mL for enzyme concentration. The rationale for such selection includes the optimal temperature and pH of enzymatic hydrolysis activity, from the above results, was 50°C and pH 6, respectively; the initial lactose concentration is limited by the maximum lactose solubility in water and high lactose concentrations are often accompanied by high GOS productivity [34,37]; 5 U/mL was considered as the optimum enzyme concentration to provide maximum GOS yields; the previous study showed that GOS reached maximum after 6 h of reaction time [38]. Results on the effect of these five factors are shown in Table 2. Moreover, two linear models were postulated to fit the experimental results and two equations for the response model were obtained as illustrated below.

**Table 2.** Experimental results for the production of GOS, GOS yield, and lactose conversion by the β-galactosidase

Run	Lactose (g/L)	pH	Tem (°C)	Time (h)	Enzyme (U/mL)	GOS (g/L)	Y_GOS_ (%)	C_lac_ (%)
1	300	5	30	4	3	160.09	53.36	46.64
2	300	6	40	10	6	99.53	33.18	66.82
3	300	7	50	20	12	80.01	26.67	73.33
4	400	5	30	10	6	174.69	43.67	56.33
5	400	6	40	20	12	128.54	32.14	67.86
6	400	7	50	4	3	122.88	30.72	69.28
7	500	5	40	4	12	177.16	35.43	64.57
8	500	6	50	10	3	185.41	37.08	62.92
9	500	7	30	20	6	165.46	33.09	66.91
10	300	5	50	20	6	92.38	30.79	69.21
11	300	6	30	4	12	97.23	32.41	67.59
12	300	7	40	10	3	112.09	37.36	62.64
13	400	5	40	20	3	138.86	34.72	65.28
14	400	6	50	4	6	148.17	37.04	62.96
15	400	7	30	10	12	132.59	33.15	66.85
16	500	5	50	10	12	160.88	32.18	67.82
17	500	6	30	20	3	237.81	47.56	52.44
18	500	7	40	4	6	263.89	52.78	47.22

**Note**: GOS is short for galacto-oligosaccharides.

Y1=36.14–4.18 × A-1.37 × B-4.06 × C + 2.03 × D-2.91 × E

**Figure 4.**
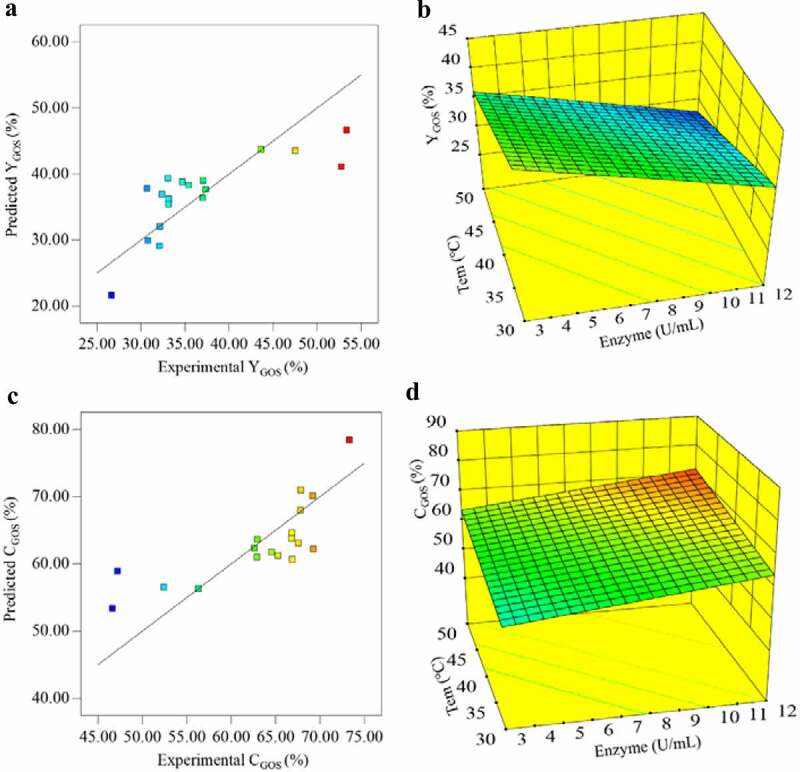
Effect of temperature and enzyme concentration on GOS synthesis: (a) Correlation between model-predicted values and experimental values of Y_GOS_ (%). (b)Three-dimensional surface plots for Y_GOS_ (%) in response to temperature and enzyme concentration. (c) Correlation between model-predicted values and experimental values of C_GOS_(%). (B)Three-dimensional surface plots for C_GOS_(%) in response to temperature and enzyme concentration

Y2 = 63.86 + 4.18 × A + 4.06 × B-0.41 × C − 2.03 × D + 2.91 × E

where Y1 was the response of GOS yield and Y2 was the response of lactose conversion, A, B, C, D, and E represented enzyme concentration, pH, temperature, initial lactose concentration, and reaction time, respectively. As shown in Figures 4A and Figure 4C, the experimental results are well in agreement with their respective predicted values obtained using the mathematical models. The correlation coefficients for the two equations were 0.93 and 0.95, respectively, and the model was statistically significant (p = 0.0303), indicating that the vast majority of the variables could be explained by this model.

Among the parameters analyzed, the response surface was significantly impacted by enzyme concentration (p = 0.0234) and temperature (p = 0.0290). Some studies have shown that the enzyme concentration had an important influence on the transgalactosylation reaction [37, 39]. The effect of temperature and enzyme concentration on the GOS synthesis can be better understood by observing the three-dimensional surface plots shown in Figure 4b-d.

By limiting these five factors (temperature, enzyme concentration, pH, initial lactose concentration, and reaction time), the maximum GOS yield was predicted by the Design Expert software (Table S1 and Table S2). As shown in Table S2, the number 1 represented the maximum production of GOS was 252.8 g/L (50.56% of the total sugar) at 30°C, pH 5.08, 500 g/L of initial lactose concentration, 3.03 U/mL of enzyme concentration, and 4 h reaction time. This yield can be considered relatively high compared to the reported yields obtained with other β-galactosidases without protein engineering as shown in Table 1.

In particular, the GOS yield obtained in this study was higher than that of Palai et al. (39% of the total sugar) who also used purified β-galactosidases derived from *B. circulans* [40]. Although the GOS yield obtained in this work was lower than that of Hung et al. (63% of the total sugar) in which recombinant β-galactosidases were derived from *Bifidobacterium infantis*, the concentration of GOS in our work was up to 252.8 g/L, which was 33% higher than their results (190 g/L) [41]. In addition, the reaction temperature was 30°C lower than the temperature used in their work, leading to more energy saving. In recent years, certain strategies have been used to improve the GOS yield, [42] predicted the key residues to transglycoside interactions of β-galactosidase from *Aspergillus oryzae*; then, a double mutant N140C/W806F was constructed and the resultant GOS yield was 59.8%, which represented substantial improvements over that of the wild type (35.7%) [42]. In another study, an intelligent double-hydrophobic amino acid scanning strategy was employed to predict key residues forming the glycan-binding site (−1 subsite) of β-galactosidase; two mutants C510V and H512I significantly improved GOS synthesis efficiency, with 59.1% for C510V and 51.5% for H512I under optimal conditions [43].

## 4. Conclusions

In this study, the characteristics of a novel β-galactosidase GALA from *B. circulans* were examined and demonstrated that the enzyme could effectively hydrolyze β-glucosides with higher transgalactosylation capacity at a lower reaction temperature and higher GOS yield than some previous studies. The optimal pH and temperature for the enzyme was 6.0°C and 50°C, which allows this enzyme to hydrolyze lactose in milk products, thereby overcoming the complications of lactase deficiency. Given the fact that the synthesis of GOS is affected by various parameters including temperature, pH, enzyme concentration, reaction time and lactose concentration, response surface methodology was employed to simultaneously evaluate the influence of these parameters and optimize the conditions for the maximum yield of GOS. The yield of GOS was approximately 50.56% of the total sugar at 30°C, pH 5.08, 3.03 U/mL of enzyme concentration, 4 h reaction time, and 500 g/L of lactose concentration, indicating potential applications of this novel enzyme in the food industry. Nonetheless, the expression level of the enzyme in this work is still not ideal, and more work is warranted to improve its expression or obtain higher transgalactosylation activity through protein engineering.

## Supplementary Material

Supplemental Material
