# Supplementary material for: β-galactosidase GALA from Bacillus circulans with high transgalactosylation activity
Source: Bioengineered. 2021 Nov 1;12(1):8908–19. doi: 10.1080/21655979.2021.1988370 (PMC8806947; doi:10.1080/21655979.2021.1988370)
Supplement: Supplemental Material [file KBIE_A_1988370_SM6995.docx]

**Supplementary Table S1**. Constraints that produce the maximum GOS yield

| Factor | Goal | Lower Limit | Upper Limit |
| --- | --- | --- | --- |
| Enzyme | In range | 3 | 12 |
| pH | In range | 5 | 7 |
| Temperature | In range | 30 | 50 |
| Lactone | In range | 300 | 500 |
| Time | In range | 4 | 20 |
| Y_GOS_ | Maximum | 0 | 100 |

**Supplementary Table S2**. The maximum production of GOS predicted by RSM

| Number | Enzyme  (U/mL) | pH | Temperature  (℃) | Lactone  (g/L) | Time  (h) | Y_GOS_  (%) |
| --- | --- | --- | --- | --- | --- | --- |
| 1 | 3.03 | 5.08 | 30.00 | 500.00 | 4.00 | 50.56 |
| 2 | 3.00 | 5.00 | 30.00 | 499.95 | 4.76 | 50.41 |
| 3 | 3.06 | 5.00 | 30.55 | 499.88 | 4.01 | 50.41 |
| 4 | 3.00 | 5.00 | 30.00 | 498.54 | 4.72 | 50.40 |
| 5 | 3.01 | 5.06 | 30.00 | 485.70 | 4.00 | 50.30 |
| 6 | 3.00 | 5.00 | 30.03 | 479.96 | 4.00 | 50.27 |
| 7 | 3.02 | 5.07 | 30.01 | 500.00 | 5.64 | 49.97 |
| 8 | 3.00 | 5.55 | 30.00 | 499.99 | 4.03 | 49.93 |
| 9 | 3.00 | 5.08 | 30.00 | 500.00 | 6.17 | 49.79 |
| 10 | 3.00 | 5.03 | 30.11 | 500.00 | 6.25 | 49.79 |
| 11 | 3.15 | 5.00 | 30.00 | 458.54 | 4.00 | 49.71 |
| 12 | 3.05 | 5.00 | 30.01 | 449.75 | 4.00 | 49.62 |
| 13 | 3.01 | 5.00 | 30.01 | 450.01 | 4.47 | 49.49 |
| 14 | 3.00 | 5.77 | 30.00 | 500.00 | 4.47 | 49.47 |
| 15 | 3.01 | 5.00 | 33.05 | 500.00 | 4.02 | 49.44 |
| 16 | 3.00 | 5.98 | 30.02 | 500.00 | 4.00 | 49.34 |
| 17 | 3.00 | 5.73 | 30.00 | 500.00 | 4.98 | 49.33 |
| 18 | 3.00 | 5.07 | 30.00 | 489.72 | 7.19 | 49.2 |
| 19 | 3.78 | 5.00 | 30.00 | 460.26 | 4.00 | 49.16 |
| 20 | 3.00 | 5.04 | 30.62 | 439.2 | 4.00 | 49.15 |
| 21 | 3.00 | 6.15 | 30.04 | 499.98 | 4.00 | 49.10 |
| 22 | 3.02 | 5.39 | 32.89 | 500.00 | 4.00 | 48.97 |
| 23 | 3.00 | 5.00 | 30.00 | 497.71 | 8.66 | 48.95 |
| 24 | 3.00 | 5.61 | 30.00 | 453.4 | 4.00 | 48.92 |
| 25 | 3.00 | 6.35 | 30.00 | 496.97 | 4.23 | 48.70 |
| 26 | 3.00 | 5.00 | 30.02 | 500.00 | 9.49 | 48.68 |
| 27 | 3.49 | 5.00 | 30.02 | 500.00 | 8.47 | 48.60 |
| 28 | 4.58 | 5.51 | 30.00 | 499.96 | 4.00 | 48.53 |
| 29 | 4.73 | 5.06 | 31.23 | 500.00 | 4.00 | 48.51 |
| 30 | 3.00 | 5.27 | 30.00 | 408.99 | 4.00 | 48.48 |
| 31 | 3.23 | 5.00 | 30.00 | 412.68 | 4.67 | 48.46 |
| 32 | 3.00 | 6.47 | 30.02 | 500.00 | 4.74 | 48.40 |
| 33 | 3.00 | 6.24 | 30.00 | 463.36 | 4.02 | 48.24 |
| 34 | 3.07 | 6.57 | 31.03 | 500.00 | 4.00 | 48.06 |
| 35 | 3.00 | 5.12 | 30.00 | 376.33 | 4.00 | 48.02 |
| 36 | 3.11 | 6.96 | 30.00 | 500.00 | 4.00 | 47.91 |
| 37 | 3.00 | 5.00 | 30.00 | 401.96 | 6.89 | 47.65 |
| 38 | 3.00 | 7.00 | 30.00 | 480.56 | 4.26 | 47.47 |
| 39 | 3.00 | 5.00 | 30.00 | 370.85 | 6.02 | 47.34 |
| 40 | 4.00 | 7.00 | 30.00 | 491.29 | 4.00 | 46.85 |
| 41 | 3.80 | 7.00 | 30.00 | 500.00 | 6.37 | 46.35 |
| 42 | 4.92 | 5.00 | 30.06 | 362.24 | 4.00 | 46.09 |
| 43 | 5.36 | 5.00 | 30.00 | 500.00 | 11.52 | 45.77 |
| 44 | 6.57 | 5.00 | 30.00 | 499.99 | 10.88 | 44.87 |
| 45 | 3.01 | 5.03 | 45.98 | 499.99 | 4.00 | 44.14 |
| 46 | 3.01 | 6.86 | 30.00 | 301.65 | 4.00 | 44.12 |
| 47 | 3.00 | 5.00 | 30.47 | 308.01 | 12.72 | 43.43 |
| 48 | 3.02 | 5.39 | 30.00 | 428.80 | 20.00 | 42.87 |

**Supplementary Figure S1**

**

**

**Fig. S1**. Extracellular β-galactosidase activity of the culture supernatant after 2 days fermentation in the buffered methanol-complex medium.
